# Scrub Typhus and COVID-19 Coinfection Unmasking Antiphospholipid Antibody Syndrome

**DOI:** 10.7759/cureus.25008

**Published:** 2022-05-15

**Authors:** Jayaram Saibaba, Jayachandran Selvaraj, Stalin Viswanathan, Vivekanandan Pillai

**Affiliations:** 1 General Medicine, Jawaharlal Institute of Postgraduate Medical Education and Research, Pondicherry, IND; 2 General Internal Medicine, Jawaharlal Institute of Postgraduate Medical Education and Research, Pondicherry, IND

**Keywords:** pulmonary embolism, antiphospholipid antibody syndrome, coinfection, covid-19, scrub typhus

## Abstract

Scrub typhus is an acute febrile disease caused by *Orientia tsutsugamushi* with a clinical course varying from mild to fatal. Vascular thrombosis is rare in scrub typhus. There is an increased risk of thrombotic events in Coronavirus disease 19 (COVID-19). We report a young diabetic female who presented with pulmonary embolism, followed by deep venous thrombosis (DVT) and was found to have coinfection with scrub typhus and COVID-19 with underlying antiphospholipid antibody syndrome.

## Introduction

Scrub typhus or tsutsugamushi disease is an acute febrile illness (AFI) caused by *Orientia tsutsugamushi* of the Rickettsiae family [1]. The disease is characterized by fever, headache, rash, and lymphadenopathy, mimicking other acute febrile illnesses such as leptospirosis, enteric fever, and dengue. Encephalitis, acute respiratory distress syndrome (ARDS), acute kidney injury (AKI), and myocarditis are examples of severe manifestations [2]. Both arterial and venous disorders have been rarely described in scrub typhus [1]. Coronavirus disease 19 (COVID-19) caused by the severe acute respiratory syndrome coronavirus 2 (SARS-CoV-2) has been described to cause thrombotic events in both arterial and venous circuits [3]. We report a young lady with scrub typhus and coronavirus disease 19 (COVID-19) coinfection who presented with pulmonary embolism as the initial manifestation and, on workup, was found to have antiphospholipid antibody syndrome (APS).

## Case presentation

This 34-year-old diabetic female presented to the local district hospital with a single episode of hemoptysis ~30 to 40 mL and shortness of breath at rest without orthopnea, wheezing, pleurisy, or fever. She had completed a six-month course of antituberculous therapy five years ago. Her diabetes was detected 18 months back, and she had been prescribed metformin 500 mg BID. She had been hemodynamically stable at that time. COVID-19 RT-PCR was negative. Computed tomography (CT) did not show an aneurysm, cavity, or other evidence of tuberculosis or COVID-19. Sputum AFB and GeneXpert were negative. She had been treated symptomatically. In the hospital, she was documented to have a fever and polyarthralgia. Laboratory investigations revealed thrombocytopenia (65 × 109/L) with normal renal and liver function tests. Fever workup showed Scrub IgM positivity (Inbios, Scrub typhus Detect IgM ELISA), and sterile cultures of blood, urine, and sputum. On day 2, she had developed pain and swelling of the left lower limb, and a compression ultrasonogram revealed a right-sided femoral deep venous thrombosis (DVT). In view of thrombocytopenia and thrombosis, disseminated intravascular coagulation (DIC) due to scrub typhus has been considered. There were no schistocytes in the peripheral smear. aPTT, fibrinogen, and D-dimer were not available in that hospital. An ultrasonogram of the abdomen did not show chronic liver disease. The patient was treated with analgesics, anticoagulation (low molecular weight heparin 40 mg subcutaneously twice daily ×5 days and warfarin 5 mg OD), ceftriaxone (2 g IV OD ×5 days), and doxycycline (100 mg BID ×14 days). There was one episode of melena on day 5, which was attributed to thrombocytopenia and anticoagulation. No blood products had been transfused. The lady was referred to our institution on day 15 for further evaluation of thrombocytopenia (values of 65, 73, 77.5, and 86.2 × 109/L over two weeks).

**Figure 1:**
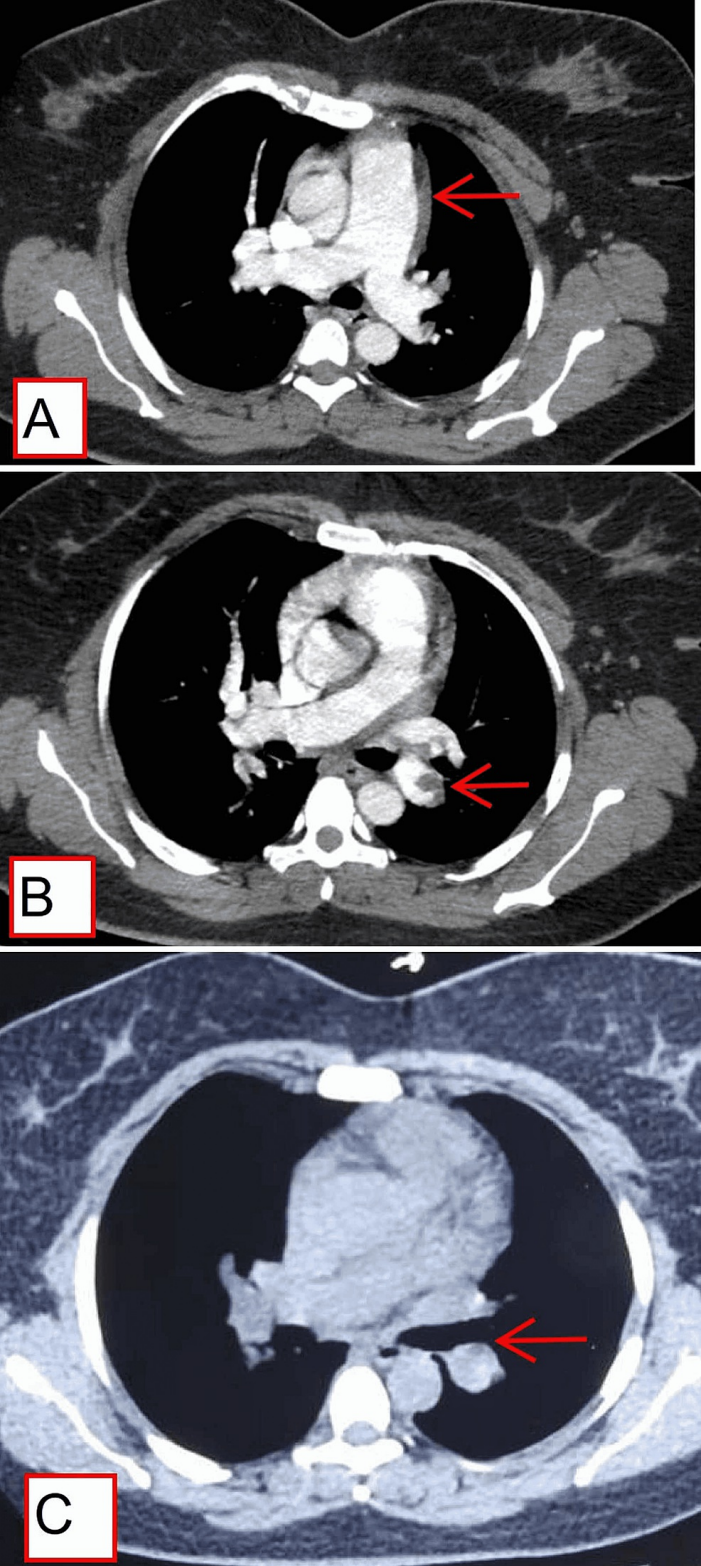
CT angiography and CT thorax findings (A) CT angiography shows dilated main pulmonary artery, (B) CT angiography shows filling-defect in the left descending pulmonary artery, and (C) non-contrast computed tomography (NCCT) thorax showing hypodensity in left pulmonary artery.

On examination, she was afebrile and had tachycardia (120 beats/min), normotension (110/60 mmHg), tachypnea (21 breaths/min), SpO_2_-94% @ room air, and pallor with a normal systemic examination. An electrocardiogram (ECG) showed sinus tachycardia with a right ventricular strain pattern. 2D echocardiography was normal. In view of DVT, a CT pulmonary angiogram was performed, and revealed a dilated main pulmonary artery of 3.4 cm (Figure 1A), with a partial filling defect in the descending branch of the left pulmonary artery (Figure 1B), extending into the superior segmental branch. The non-contrast CT thorax from the previous hospital, when reviewed, also showed a hypodensity in the left pulmonary artery (Figure 1C). Scrub IgM and COVID RT-PCR done at the time of admission to our hospital were positive. She had not been vaccinated for COVID-19. Other investigations are listed in Table 1. She was shifted to the COVID-19 hospital on our campus and managed with enoxaparin and warfarin. CT did not show any evidence suggestive of COVID infection (CORADS-1). She required minimal oxygen through nasal prongs (2 L/min) for ~24 hours. In view of the probable asymptomatic COVID-19 infection, dexamethasone was not administered. She was moved to the general ward ten days later, following mandatory institutional quarantine (asymptomatic COVID-19 with another medical condition requiring admission). In view of venous thromboembolism and a past history of second-trimester abortion, APS was suspected. Tests for APS were performed in a private lab since we did not have facilities for the same, with antinuclear antibody (ANA) 3+ positivity (homogeneous pattern), anticardiolipin IgM antibody positivity (>40), and beta 2 glycoprotein-1 IgM and IgG elevated levels (Table 1). She could not afford an ANA blot at this time.

**Table 1:** List of investigations during hospital stay

Investigations	Day 16	Day 26	Day 28
Hemoglobin (g/L)	91	92	97
Total leukocyte counts (4–11 × 10^9^/L)	6	7.48	8.13
Platelets (150–450 × 10^9^/L)	99	90	122
Mean corpuscular volume (78–98 fL)	70.1	73.4	
Ferritin (10–120 ng/mL)		15.1	
Serum iron (60–180 μg/mL)		25	
Transferrin saturation (20–50%)		6	
Prothrombin time (test/control)	13.2”/11.8”		
International normalized ratio	1.11	1.5	1.7
aPTT (test/control)	65.8”/31.5”		
Fibrinogen (200–400 mg/dL)	335		
Thrombin time (test/control)	16.2”/14”		
d-Dimer (0.21–0.36 μg/mL)	1.63		
HbA1c %			8.2
Antinuclear antibody		3+ Homogenous	
Lupus anticoagulant		Detected	
Beta 2 glycoprotein 1-IgM (>20 RU/mL)		93.3	
Beta 2 glycoprotein 1-IgG (>20 RU/mL)		111.34	
Anticardiolipin IgM (GPL U/mL)		64.67	
Anticardiolipin IgG (GPL U/mL)		Negative	
HBsAg	Negative		
Anti HCV	Negative		
HIV 1 and 2	Nonreactive		
Urinalysis	Albumin 1+		
Dengue IgM ELISA and NS1	Negative		
Chikungunya IgM and PCR	Negative		
Scrub IgM ELISA	Positive		
TSH (0.5–5.5 IU/mL)	2.2		

The patient was advised of lifelong anticoagulation and discharged on day 30 of illness, with follow-up visits to be scheduled by teleconsultation. On follow-up two weeks later by teleconsultation, her INR was 2.3, and she had no new cardiorespiratory symptoms or fever. The patient gave written informed consent for the publication of her details along with the images.

## Discussion

Scrub typhus is a common cause of acute febrile illness in most parts of Asia; along with multi-system manifestations, it is characterized by an eschar at the site of a mite bite. The infection responds dramatically to antibiotics such as doxycycline [2]. In India, it has been described in both urban and rural areas and has been reported in most states [4]. Systemic vasculitis and perivasculitis are the main pathologic findings in scrub typhus and are caused by the proliferation of *O. tsutsugamushi* in the endothelial cells of the microvascular system [2]. One report suggested the concept that acute infections were associated with a transient increase in the risk of vascular events [5]. The exact mechanism by which acute infection or inflammation affects the risk of vascular events is unknown. Coagulation changes in scrub typhus infection are characterized by inflammation-induced coagulopathy with coagulation activation, antithrombin III and soluble thrombomodulin depletion, and an increase in proinflammatory cytokines (TNF- alpha, IL-1beta, IL-6), tissue factor, and C-reactive protein [6]. Markers of coagulation such as thrombin-antithrombin complexes and soluble tissue factor levels were elevated in patients with scrub typhus [6]. Vascular events reported in scrub typhus include myocardial infarction [7], strokes, digital gangrene [8], DVT [9], cerebral venous thrombosis [10], and pulmonary venous thromboembolism [1]. In the patient from Korea, pulmonary embolism developed one week following discharge, but in our case, she presented with embolism and was found to have fever and thrombocytopenia [1].

Autopsy studies in COVID-19 decedents showed microvascular and macrovascular thrombosis involving both the arterial and venous circulation [3]. The angiotensin-converting-enzyme 2 receptors, to which the COVID-19 virus has affinity, are found in high density in the arteries and veins. Endotheliitis, platelet activation, neutrophil extracellular traps (NETs), activated platelets, and increased von Willebrand factor levels lead to thrombin and fibrin formation. Microvascular thrombosis containing platelet-thrombi has been reported in capillaries, arterioles, and venules of all major organs. Venous thromboembolism, myocardial infarction, and strokes have been reported in many studies involving patients with COVID-19 [3]. In our patient, the initial RT-PCR was negative in the referring hospital but positive on arrival at our institution. COVID-19 coinfections with bacteria, fungi, and other viruses have been described. Scrub typhus coinfection has not been previously reported [11]. So, both organisms can cause endothelial dysfunction and activation of the coagulation cascade that could lead to venous thromboembolism.

Anemia may be coupled with reactive thrombocytosis that could predispose to thrombosis, but that was not seen in our case. Metformin is also a risk factor for anemia, but her mean corpuscular volume was suggestive of microcytic anemia. Thrombocytopenia was probably contributed by both infections and APS. Whether it was systemic lupus-related APS could not be confirmed. Thrombocytopenia is one of the predictors of APS-related thrombotic manifestations [12]. Thrombocytopenia is common in scrub typhus and less common in COVID-19-related coagulopathy [3]. Infections may elevate levels of antiphospholipid antibodies. Abdel-Wahab et al. reviewed 293 cases of infection associated with APS with a mean age of 34 years [13]. Their review described patients who had infection followed by elevated antiphospholipid antibodies (persistent or transient) with or without thromboembolic manifestations. Viruses (human immunodeficiency virus and hepatitis C) were the most common, followed by bacteria. Rickettsia africae and Rickettsia typhi have been reported, while there are no reports of scrub typhus. Since this review was published in 2016, COVID-19 did not merit mention [13].

## Conclusions

We report a patient with an antiphospholipid syndrome that was unmasked by scrub typhus and asymptomatic COVID-19 coinfection and presented with pulmonary embolism and DVT as the initial manifestation of a coinfection. Either of these illnesses may predispose to thrombosis and coagulopathy, and a coinfection could theoretically increase this risk. In the time of COVID-19, it is always mindful to remember the commoner causes of fever and thrombosis in the tropics.
